# Double Whammy: A Case of Concurrent Alcohol Use and Hereditary Hemochromatosis Leading to Avascular Necrosis of the Femur

**DOI:** 10.7759/cureus.18067

**Published:** 2021-09-17

**Authors:** Christine E Albers, Janet Albers, Amit Sapra, Priyanka Bhandari, Eukesh Ranjit

**Affiliations:** 1 Family and Community Medicine, Southern Illinois University School of Medicine, Springfield, USA; 2 Family Medicine, Southern Illinois University School of Medicine, Springfield, USA

**Keywords:** avascular necrosis (avn), hereditary hemochromatosis, hip pain, rare association, idiopathic osteonecrosis, hemochromatosis, osteonecrosis, ischemic necrosis, double whammy, alcoholism

## Abstract

Avascular necrosis (AVN) of the femur is due to damage to the vasculature of the bone and can include a multitude of causes including medications, alcohol intake, hemoglobinopathies, thrombophilias, and connective tissue disorders, among others. Hereditary hemochromatosis is not a common cause of AVN but should be considered prior to labeling such cases as idiopathic. If a patient has symptoms of persistent hip pain and plain radiography has been unremarkable, one should proceed with magnetic resonance imaging (MRI) of the affected joint due to its sensitivity in detecting osteonecrosis. We present a case report of a 54-year-old male patient with a significant history of alcohol intake and a diagnosis of hereditary hemochromatosis who presented with persistent left hip pain and further imaging revealed the presence of osteonecrosis of the femur.

## Introduction

Avascular necrosis (AVN), also known as osteonecrosis, aseptic necrosis, and ischemic necrosis, is a compromise of the bone vasculature leading to bone marrow infarction and ultimate mechanical failure [1]. In some cases, this is due to a bone or joint trauma. In other cases, the exact pathogenesis is unclear but presumed that certain risk factors affect the microcirculation that leads to necrosis of the underlying bone. This rare condition can lead to extensive morbidity if left undiagnosed and undetected. The joint destruction sets in early, making joint preservation impossible and necessitating a joint replacement. There have been case reports of the association of AVN with alcoholism and scattered case reports mentioning the association with hereditary hemochromatosis. We were, however, unable to find any case reports in the literature of a case of AVN where both these predisposing factors were present.

## Case presentation

**Figure 1:**
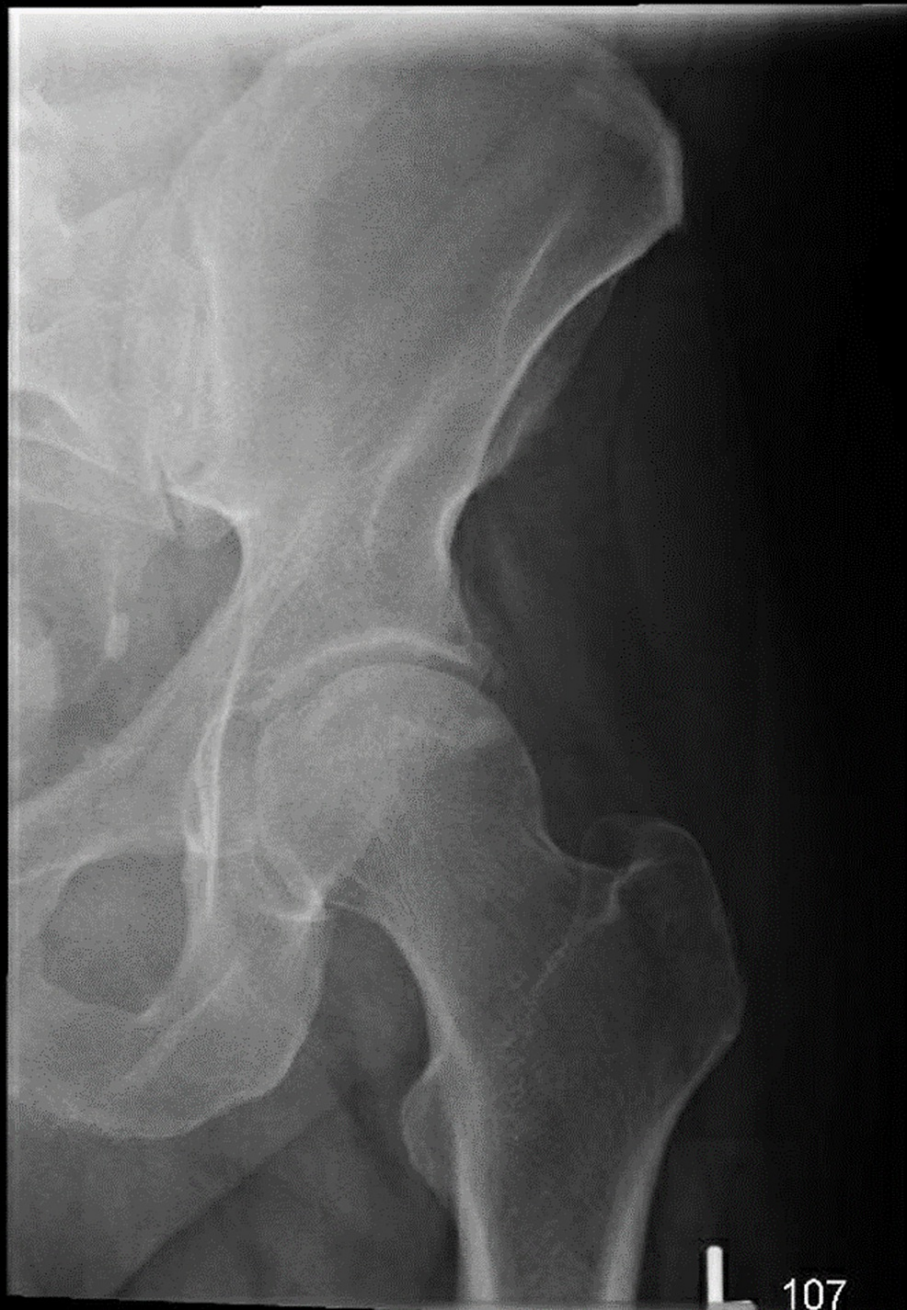
X-ray of the left hip showing that the joint spaces are maintained and there is no acute abnormality.

**Figure 2:**
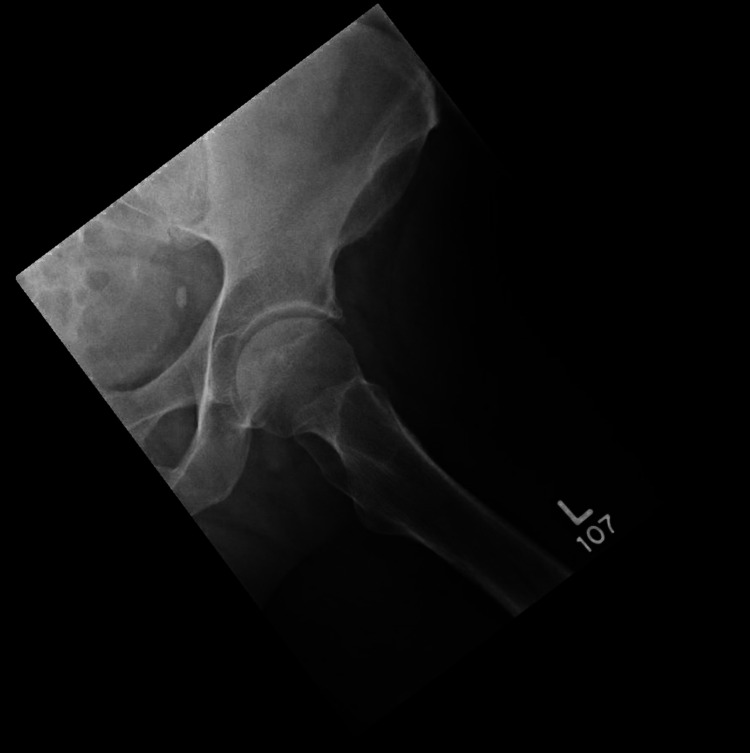
X-ray of the left hip showing that the joint spaces are maintained and there is no acute abnormality.

Our patient is a 54-year-old Caucasian male with a past medical history of hemochromatosis (requiring bi-monthly therapeutic phlebotomies), hypertension, depression, and obstructive sleep apnea, who presented to his primary care physician's oﬃce with persistent left hip pain worsening with weight-bearing. He complained of feeling clicks and pain on the anterolateral left hip area and stated that the pain radiated down to the anterior thigh, anterior knee, and dorsal foot and toes. He denied any back pain but did complain of numbness in bilateral toes. He also complained of weakness while dorsiﬂexing his left foot. He had done physical therapy and alternate heat and ice therapy with no relief. He stated he received only temporary relief with the prescribed nonsteroidal and muscle relaxants. He denied fever, chills, rigors, malaise, or poor appetite. Before presenting to his primary care physician, the patient was seen by a sports medicine specialist, and had been diagnosed with left gluteus medius tendinopathy and left trochanteric bursitis. He had also received X-rays of the left hip, which were negative for arthritis, fracture, dislocation, or another acute osseous process, as well as point of care ultrasound of the left hip, which showed no signiﬁcant ﬁndings (Figures 1, 2).

Physical exam revealed a limp on the left lower extremity with ambulation. There was moderate tenderness to palpation on the anterolateral left hip joint and minimal tenderness to palpation on the greater trochanter of the left hip. There was no tenderness to palpation on the sacroiliac joint or in the paraspinal muscle area. Flexion, adduction, internal rotation (FADIR) test was very limited due to severe pain. Log roll test was positive. The flexion, abduction, external rotation (FABER) test was somewhat limited with moderate pain. The straight leg raise test was positive on the left. The left lower extremity strength was 4/5 with dorsiﬂexion of ankle/first toe and extension of the knee, and the right lower extremity strength was 5/5.

The patient was continued on his current pain management regimen and given a prescription for oblique crutches and a quad cane to unload the weight-bearing on his left hip. An urgent magnetic resonance imaging (MRI) of the left hip and lumbar spine without contrast was ordered for further diagnosis. Unfortunately, the MRI examination of the left hip was incomplete due to the patient's inability to tolerate the procedure due to worsening pain.

**Figure 3:**
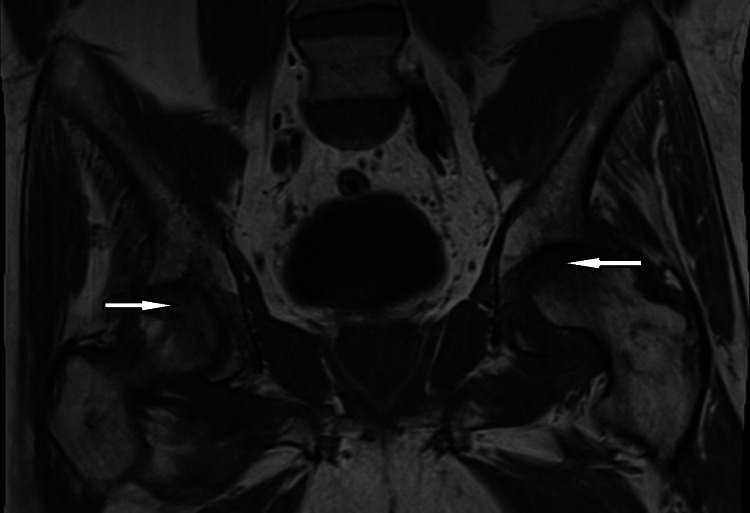
MRI of the left hip showing evidence of marked degenerative changes at the hip joints. It also demonstrates the presence of osteonecrosis of both femoral heads with partial collapse of the left femoral head (white arrows).

The images obtained demonstrated marked degenerative changes at the hip joints. They also demonstrated the presence of osteonecrosis of both femoral heads with partial collapse of the left femoral head and surrounding bone marrow edema (Figure 3).

An urgent orthopedic consult determined a diagnosis of left hip degenerative arthrosis secondary to avascular necrosis. Ultimately, the patient underwent left total hip arthroplasty (primary, cementless) via a direct anterior approach. The patient tolerated the procedure well, and his postoperative period was unremarkable. He was discharged home with self-care and advised physical therapy and occupation therapy.

In evaluating the possible underlying risk factors for avascular necrosis for this patient, the possible relationship to his diagnosis of hereditary hemochromatosis was considered. In addition, there was a notable history of frequent alcohol use in the patient. He was a non-smoker and denied any illicit drug usage. There was no history of diabetes, hyperlipidemia, pancreatitis, or hyper-homocysteinemia. As stated above, there was no history of trauma, radiation exposure, or any history of decompression disease. There was no personal or family history of any connective tissue disorder, including lupus, hemoglobinopathies, thrombophilia, or hereditary osteonecrosis. He was not on any form of systemic steroids, heparin, bisphosphonates, or other anti-resorptive agents.

## Discussion

Epidemiology

Avascular necrosis (AVN) is also known as osteonecrosis (ON), ischemic necrosis, or aseptic necrosis, and has an incidence of 300,000-600,000 cases in the general population in the early 2000s in the United States [1]. Males are affected three times more commonly than females, and the average age of the affected patient is around the mid-40s [1]. Most commonly, the proximal end of the femur is affected.

Etiology

The etiology of AVN can be classified into two categories, traumatic and non-traumatic. The most common cause is a result of trauma secondary to a displaced hip fracture or dislocation. However, various non-traumatic causes also contribute to the etiology of AVN, including alcohol use, tobacco use, chemotherapy, HIV, autoimmune disorders, and coagulation disorders such as sickle cell disease, thalassemia, thrombophilia, and chronic use of corticosteroids [1,2]. Both alcohol and corticosteroids are dose-dependent, with larger amounts for an extended period of time, increasing the risk of AVN. It is estimated that 80% of cases of AVN not related to trauma are caused by alcohol and corticosteroid use [3]. Hemochromatosis does not directly correlate to AVN; however, it should be noted that those with hemochromatosis are at risk for hip arthropathy, leading from an increased risk of AVN [4,5]. Before labeling avascular necrosis as idiopathic, iron studies should be strongly considered to rule out hemochromatosis [4].

Pathogenesis

Although the etiology may vary, in general, AVN is brought on by a disruption in circulation in the subchondral bone, resulting in ischemia to the femoral head and bone marrow, destroying osteocytes [6]. The resulting necrosis of new and existing bone creates an inflammatory response that leads to resorption of acellular bone, which propagates demineralization and loss of structural integrity, ultimately causing bone collapse and joint destruction [3,6].

Symptoms

In its earliest stages, patients may remain asymptomatic for weeks or even months. A typical symptom in a patient with AVN is pain, which is insidious in onset and worsens with activity and weight-bearing [7]. As the bone destruction progresses, patients may experience pain in the anterior hip and groin, which is worse with movement and weight-bearing exercises and radiate to the thigh and knee [2]. The patient may also develop a limp [2]. AVN should be considered in a patient with prolonged hip pain with a normal X-ray [8].

Diagnosis

MRI is the imaging modality of choice [9]. However, referring physicians need to be able to assess MRI safety, and compatibility of medical devices to keep patients safe [10]. It is worth noting that 18.75% of new presentations are diagnosable only with MRI and may be easily missed [2]. MRI changes include well-demarcated and homogenous focal lesions on T1-weighted images with a single-density line separating normal and ischemic bone and a second high-intensity line on T2-weighted images, known as the double-line sign is pathognomonic for AVN [3]. The imaging findings can be classified using the Ficat and Arlet systems [1,3]. Normal X-rays may be falsely normal in the early stages [11]. Lab testing has minimal value in the diagnosis of AVN.

Management

The method of management depends on the level of severity of necrosis. Non-surgical management is preferred in mild cases and includes physical therapy, nonsteroidal anti-inflammatory drugs (NSAIDs), acetaminophen, and short courses of opioids. More studies are needed to fully assess the value of anticoagulants, cholesterol-lowering medications, bisphosphonates, and hyperbaric oxygen. Core decompression is one surgical treatment that provides a pathway for new blood vessels to promote healing by drilling holes in the affected bone. This can be combined with bone grafting to stimulate healthy bone growth as well. Tantalum rod insertion is an alternative to bone grafting that provides structural support and bone growth due to its osteoconductive texture. Osteotomies are another option for prolonging joint replacement and involve transposing the necrotic area from a weight-bearing to a non-weight-bearing area of the hip joint [1,3,9,12].

Complications and prognosis

As many patients do not present until the disease has progressed to more advanced stages, total joint replacement is a common complication of AVN [9]. Of symptomatic cases, 80%-85% will result in the collapse of the femoral head within two years, resulting in joint replacement [3]. As most joint replacements last 15 years, younger patients may require a revision [2]. Following the resolution of AVN, most patients will develop osteoarthritis, but this can be prevented by keeping a healthy weight and remaining active.

## Conclusions

This case illustrates that physicians should keep a high suspicion of avascular necrosis in a patient with persistent joint pain and with alcohol intake. An appropriate social history of alcohol use may elucidate underlying risk factors. In addition, one must keep in mind the rare possible association of AVN with hereditary hemochromatosis in patients with persistent joint pain. Evaluation for iron balance might be a consideration before we label those cases as idiopathic.
